# Perioperative Blood Transfusion and Delirium after Total Knee or Hip Arthroplasty: Retrospective Analysis

**DOI:** 10.3390/jpm14060576

**Published:** 2024-05-28

**Authors:** Saeyeon Kim, Tak-Kyu Oh, In-Ae Song

**Affiliations:** 1 Department of Anesthesiology and Pain Medicine, Seoul National University Bundang Hospital, Seongnam 13620, Republic of Korea; cecillia2@gmail.com; 2 Department of Anesthesiology and Pain Medicine, College of Medicine, Seoul National University, Seoul 01811, Republic of Korea

**Keywords:** total knee arthroplasty, total hip arthroplasty, postoperative delirium, blood trans fusion

## Abstract

We investigated the type of blood component transfusion associated with increased postoperative delirium. Adult patients who underwent total knee arthroplasty (TKA) or total hip arthroplasty (THA) between 2017 and 2022 were included. Delirium was evaluated and treated within two days after surgery. A total of 6737 patients (4112 TKA/2625 THA) were retrospectively studied; 2.48% of patients in the TKA (n = 102) and THA (n = 65) groups had postoperative delirium. The blood transfusion (BT) and non-BT groups had similar percentages of patients who experienced postoperative delirium (3.34 vs. 2.35%, *p* = 0.080). In the multivariable logistic regression model, BT was not associated with postoperative delirium—adjusted odds ratio (aOR): 1.03, confidence interval (CI): 0.62, 1.71; *p* = 0.917. Moreover, transfusion of packed red blood cells (*p* = 0.651), platelets (*p* = 0.998), and cryoprecipitate (*p* = 0.999) were not associated with delirium. However, transfusion of fresh frozen plasma was associated with a 5.96-fold higher incidence of delirium—aOR: 5.96, 95% CI: 2.72, 13.04; *p* < 0.001. In conclusion, perioperative BT was not associated with postoperative delirium in patients who underwent TKA or THA. However, FFP transfusion was associated with an increased incidence of postoperative delirium.

## 1. Introduction

Total joint arthroplasty (THA) is one of the most effective medical treatments for hip and knee osteoarthritis. It significantly lowers pain, restores function, and improves quality of life [1]. Total knee arthroplasty (TKA) and total hip arthroplasty (THA) are the most common surgical procedures for treating osteoarthritis [2,3]. THA and TKA rates have significantly increased and are predicted to continue rising in the United States and Europe [4,5]. As a result, THA and TKA are becoming a bigger public health risk.

Transfusion rates for TKA vary from 3 to 67%, whereas those for THA vary from 4 to 68%, because there is no clear consensus on the appropriate indications for transfusion [6]. While transfusing blood components can treat anemia following perioperative blood loss, it carries risks such as blood-borne infections, allergic reactions, and transfusion reactions, as well as significant costs to the healthcare system [7]. Furthermore, several studies have shown that perioperative blood transfusions can trigger inflammation and are independent risk factors for postoperative delirium in older patients undergoing surgery [8,9]. With a reported prevalence of 2.21%, delirium is a serious consequence that might slow patient recovery and increase hospital stay after THA and TKA [10]. Some risk factors such as opioid use, benzodiazepine use, and general anesthesia have been reported [10]. However, the relationship between perioperative blood transfusion and postoperative delirium in patients who underwent TKA or THA is not fully understood.

Therefore, we aimed to determine the type of blood component transfusion that increases the incidence of postoperative delirium. We hypothesized that blood transfusion might increase the risk of delirium in patients who had undergone TKA or THA.

## 2. Materials and Methods

### 2.1. Ethical Statement

This retrospective cohort study was approved by the Institutional Review Board (IRB) of our institution (IRB approval number: B-2307-843-111). The requirement for informed consent was waived by the IRB. This manuscript adheres to the Strengthening the Reporting of Observational Studies (STROBE) guidelines.

### 2.2. Data Source and Study Population

We included 6737 cases from 5195 consecutive patients at a single tertiary academic hospital that employs BESTCare [11] for electronic health records. We retrospectively evaluated adult patients (18 years or older) who had undergone elective TKA, THA, or revision TKA/THA under general or neuraxial anesthesia between 1 January 2017, and 31 December 2022. We excluded patients who had died in the hospital postoperatively and those who lacked pertinent medical information. Diagnoses and procedures were based on the International Classification of Diseases, Ninth Revision, Clinical Modification (ICD-9-CM).

### 2.3. Study Endpoints (Diagnosis of Delirium)

The primary endpoint was the diagnosis of delirium. We extracted data on delirium observed and treated within two days after surgery, based on the evaluation of psychiatrists or medications for treatment, such as IV or oral benzodiazepine (lorazepam and alprazolam) or antipsychotics (haloperidol, quetiapine, olanzapine, and risperidone) at low doses. 

### 2.4. Potential Confounders

We extracted patients’ baseline medical information, including age, sex, preoperative hemoglobin (Hb) level, medical history, American Society of Anesthesiologists (ASA) physical status, and body mass index (BMI), from the electronic health record system. Preoperative anemia was defined if Hb was recorded below the following values: 12.0 g/dL for women, 13.0 g/dL for men [12]. We used standard BMI categories: BMI groupings of less than 18.5 kg/m^2^ (underweight), 18.5–24.9 kg/m^2^ (healthy), 25.0–29.9 kg/m^2^ (overweight), and 30.0 kg/m^2^ or above (obese) [13]. Medical history included diabetes mellitus, hypertension, carotid artery disease, cerebrovascular disease, liver disease, dementia, chronic kidney disease, and mental illness. We searched for preoperative assessments recorded by anesthesiologists and admission records by ward nurses. In this study, we defined mental illness as a diagnosis of depressive, bipolar, or psychotic disorder, regardless of medication. For chronic kidney disease, we only included patients with end-stage renal disease who were undergoing dialysis. Regarding surgery-related information, we collected data on whether patients received continuous intravenous infusion of magnesium sulfate during surgery, duration of anesthesia (in minutes), type of anesthesia (general or regional), adjuvant sedatives used (dexmedetomidine, propofol, or midazolam) in addition to the primary anesthetics, postoperative transfer (to the general ward or intensive care unit), year of surgery, type of surgery, and whether each surgery was the first, second, or a revision. One group of patients underwent a second TKA or THA on the other side, with an interval of one week between the procedures. An anesthesiologist or registered nurse recorded the estimated blood loss during surgery. In addition, we collected data on whether a patient had received any type of blood transfusion—red blood cells, fresh frozen plasma (FFP), platelets, or cryoprecipitate—on the day of surgery. 

### 2.5. Statistical Analysis

All statistical analyses were performed using the Statistical Package for the Social Sciences Statistics for Windows (version 25.0; IBM Corp., Armonk, NY, USA), and statistical significance was set at *p* < 0.05. The baseline characteristics of the patients were presented as mean values with standard deviations (SDs) for continuous variables and as numbers with percentages for categorical variables. Multivariable logistic regression modeling was used to identify factors associated with postoperative delirium after TKA or THA. To study the effect of transfusion on delirium incidence, we included essential covariates such as estimated blood loss and anemia and other covariates that had a *p*-value larger than 0.1 from the univariable logistic regression model. In Model 1 of the multivariable logistic regression analysis, we included patients who received any type of blood component in the blood transfusion group and compared them with those who did not receive any blood transfusions. Model 2 differentiated the blood transfusion group into blood component groups for comparison. We performed subgroup analyses for age, sex, surgery type, and anemia status.

## 3. Results

### 3.1. Study Population

A total of 6737 patients were enrolled in this study, of whom 4112 had undergone TKA and 2625 had undergone THA between January 2017 and December 2022. The demographic and clinical data of the enrolled patients are shown in Table 1. While 2.48% of the TKA (n = 102) and THA (n = 65) groups had postoperative delirium, more patients in the THA group received postoperative blood transfusion and experienced a larger amount of estimated blood loss than those in the TKA group. Patients in the TKA group were significantly more anemic than those in the THA group.

### 3.2. Blood Transfusion 

Table 2 compares the characteristics of patients who received any type of blood component postoperatively and those who did not. The blood transfusion (BT) group was younger, had greater estimated blood loss, and had more patients with anemia than the non-BT group, whereas there was no difference between the two groups in the incidence of delirium. 

Along with variables of interest, including transfusion, estimated blood loss, and anemia, we selected factors with a P-value greater than 0.1 from the univariable logistic regression model (Table S1) for analysis in the multivariable logistic regression model. Table 3 shows the results of the multivariable logistic regression analysis for delirium after TKA and THA. In multivariable model 1, the BT group was not associated with delirium after TKA and THA—adjusted odds ratio (aOR): 1.03, confidence interval (CI): 0.62, 1.71; *p* = 0.917. In multivariable model 2, RBC transfusion (aOR: 0.88, 95% CI: 0.50, 1.55; *p* = 0.651), PLT (aOR: 0.00, 95% CI: 0.00, *p* = 0.998), and CPP (aOR: 0.00, 95% CI: 0.00; *p* = 0.999) were not associated with delirium after TKA and THA. However, transfusion of FFP was associated with a 5.96-fold higher incidence of delirium (aOR: 5.96, 95% CI: 2.72, 13.04; *p* < 0.001). BMI, ASA, type of anesthesia, and sedative use were not significantly associated with the occurrence of delirium after surgery (all *p* > 0.05). 

### 3.3. Subgroup Analyses

Table 4 shows the results of the subgroup analyses for postoperative delirium according to age, sex, surgery type, and hemoglobin level. However, subgroup analyses did not show statistically meaningful results (all *p* > 0.05).

## 4. Discussion

No correlation was found between the incidence of delirium in patients who underwent TKA or THA and blood transfusions in this retrospective population-based cohort study conducted in a single institution. However, among blood products, FFP transfusion was linked to an increased risk of delirium. Our main conclusions are derived from a variety of risk variables for delirium; however, our findings also suggest that additional studies are necessary to corroborate these conclusions.

Several previous studies reported that blood transfusion was associated with an increased risk of delirium after orthopedic surgeries [9,14,15]. However, another cohort study reported no significant association between blood transfusion and postoperative delirium [16]. This lack of consistency can be attributed to variations in the threshold for the amount of blood transfused, types of blood components, indication of blood transfusion, and diverse characteristics and conditions of the targeted patients across different studies. Thus, the relationship between blood component transfusion and delirium risk in patients undergoing orthopedic surgery, especially TKA or THA, remains debatable.

Although overall blood transfusion was not associated with the risk of postoperative delirium, transfusion of FFP was associated with an increased risk of postoperative delirium in this study. Andrási et al. identified FFP transfusion as a risk factor for postoperative delirium after cardiac surgery [17]. Consistent with this, we also observed an elevated likelihood of postoperative delirium associated with FFP transfusion. One possible explanation for the development of postoperative delirium is the initiation of an inflammatory response triggered by blood transfusion; systemic inflammation could contribute to the pathogenesis of delirium by compromising the blood–brain barrier integrity [18]. Furthermore, transfusion of FFP induces a spontaneous and dose-dependent release of proinflammatory cytokines such as tumor necrosis factor-alpha (TNF-α) [19], which is related to a hyperactive form of delirium [20]. Systemic TNF crosses the blood–brain barrier, and the excessive presence of circulating TNF is associated with cognitive dysfunction [21], which can result in postoperative delirium. 

FFP was obtained through a freeze/thaw process; however, leukocyte reduction was not observed. As a result, FFP contains a large number of white blood cells and inflammatory mediators, such as mitochondrial damage-associated molecular patterns (DAMPs) and TNF-α, resulting from the rupture of cellular components. DAMPs, which are detected significantly more frequently in FFP than in other blood components, trigger an inflammatory response upon release into the plasma [22]. Receiving multiple units of FFP may expose patients to a quantity of pro-inflammatory cytokines and mediators that can exacerbate an existing inflammatory cascade caused by surgery.

Beyond blood transfusion, it is possible that the development of delirium was associated with anemia in the patient requiring transfusion. In patients with anemia, low hemoglobin concentrations lead to reduced cerebral oxygen delivery [23]. Moreover, low hemoglobin concentrations reflect an inflammatory state associated with chronic diseases [24]. Both explanations are considered possible pathological mechanisms underlying delirium. Unlike other risk factors for postoperative delirium, preoperative anemia can be corrected with medical treatments, such as tranexamic acid, iron supplements, or erythropoietin-stimulating agents. 

Previous studies demonstrated that pre-existing dementia or psychiatric disorders are associated with an elevated risk of postoperative delirium [25,26], which is consistent with our findings. Despite the well-established status of dementia as a risk factor for delirium, the underlying mechanism remains elusive. Robinson et al. explained the link between pre-existing dementia and postoperative delirium through the “threshold theory”, which posits that a decline in cognitive reserve heightens vulnerability to cognitive clinical deficit and results in delirium [27]. Some prior research proposed that neuropathophysiological changes contribute to increased susceptibility to postoperative delirium. These changes, indicative of reduced “brain reserves”, are characterized by slower cerebral metabolism, cholinergic deficiency, and inflammation [28]. 

Among the various psychiatric disorders, depression is recognized as a risk factor for postoperative delirium by the European Society of Anesthesiology [29]. Depressive symptoms might lead to underlying cerebral dysfunction, such as abnormal stress or brain inflammatory responses, which predisposes patients to an exaggerated risk of postoperative delirium [30]. Elevated cortisol levels (stress hormones) associated with depression can induce structural changes in hippocampal neurons and disrupt the hypothalamic–pituitary axis. These changes may increase susceptibility to episodes of postoperative delirium, thus providing evidence for a potential connection between depression, cortisol, and the development of postoperative delirium [31]. 

This study had several limitations. First, due to its retrospective nature, we could not identify patients with postoperative delirium using standardized and consistent assessment tools, such as the Confusion Assessment Method or the Delirium Rating Scale. We relied on observations made by physicians or nurses, consultations, and medication orders. Consequently, this might have resulted in under-recognition of the hypoactive form of delirium. Second, owing to the challenges associated with electronic medical records, we could not extract the exact quantity of blood units administered to each patient. We could not account for the potential impact of massive blood transfusions or the dose-dependent effects of blood transfusions in our analysis. 

## 5. Conclusions

In this retrospective analysis of 6737 adult patients who underwent TKA or THA, we found no association between blood transfusions and delirium incidence. Nonetheless, among blood products, FFP transfusion was associated with a higher risk of delirium. Moreover, some risk factors for postoperative delirium were identified, such as anemia, dementia, or mental illness. Our study suggests the need for limited and conservative blood transfusions to prevent delirium after TKA or THA. This also shows that FFP transfusion should only be performed after careful consideration. Nevertheless, numerous studies indicate that there is still an ongoing debate regarding this subject, emphasizing the importance of exercising caution when interpreting the findings and implementing them in a clinical setting. Further investigation should prioritize the examination of blood transfusion components and criteria, as well as explore the correlation between anemia and the potential for postoperative delirium.

## Figures and Tables

**Table 1:** Baseline information of patients.

Variable		Total N = 6737	TKA N = 4112	THA N = 2625	*p*-Value
Age, Year (Mean, SD)		66.14 (12.55)	71.76 (6.68)	57.34 (14.40)	<0.001
Sex, Female—N (%)		4922 (73.06)	3547 (86.26)	1375 (52.38)	<0.001
Delirium		167 (2.48)	102 (2.48)	65 (2.48)	0.991
Transfusion					
	Any	868 (12.88)	238 (5.79)	630 (24.00)	<0.001
	RBC	631 (9.37)	208 (5.06)	423 (16.11)	<0.001
	FFP	115 (1.71)	15 (0.36)	100 (3.81)	<0.001
	PLT	36 (0.53)	12 (0.29)	24 (0.91)	0.001
	Cryoprecipitate	2 (0.03)	1 (0.02)	1 (0.04)	0.749
Estimated Blood Loss (mL/100)		3.79 (5.49)	0.94 (1.99)	8.25 (6.20)	<0.001
Anemia		2096 (31.11)	1588 (38.62)	508 (19.35)	<0.001
MgSO_4_		1686 (25.03)	932 (22.67)	754 (28.72)	<0.001
Medical History					
	DM	1515 (22.49)	1173 (28.53)	342 (13.03)	<0.001
	HTN	3933 (58.38)	2955 (71.86)	978 (37.26)	<0.001
	CAD	613 (9.10)	483 (11.75)	130 (4.95)	<0.001
	CVD	689 (10.23)	529 (12.86)	160 (6.10)	<0.001
	Liver Disease	34 (0.50)	18 (0.44)	16 (0.61)	0.332
	Dementia	21 (0.31)	19 (0.46)	2 (0.08)	0.006
	Chronic Kidney Disease	133 (1.97)	91 (2.21)	42 (1.60)	0.078
	Mental Illness	125 (1.86)	75 (1.82)	50 (1.90)	0.811
BMI					<0.001
	Underweight	88 (1.31)	19 (0.46)	69 (2.63)	
	Healthy Weight	2411 (35.79)	1091 (26.51)	1321 (50.32)	
	Overweight	3174 (47.11)	2178 (52.97)	996 (37.94)	
	Obese	1064 (15.79)	825 (20.06)	239 (9.10)	
ASA Physical Status Classification					<0.001
	1	1077 (15.99)	331 (8.05)	746 (28.42)	
	2	4634 (68.78)	3020 (73.44)	1614 (61.49)	
	≥3	1026 (15.23)	761 (18.51)	265 (10.10)	
Anesthesia Time (min/30)		4.71 (1.35)	4.44 (1.24)	5.13 (1.41)	<0.001
Type of Anesthesia					0.304
	Regional Anesthesia	6311 (93.68)	3862 (93.92)	2449 (93.30)	
	General Anesthesia	426 (6.32)	250 (6.08)	176 (6.70)	
Use of Sedatives					
	Dexmedetomidine	1249 (18.54)	586 (14.25)	663 (25.26)	<0.001
	Propofol (R/A only)	2916 (43.28)	1649 (40.10)	1267 (48.27)	<0.001
	Midazolam	4235 (62.86)	2274 (55.30)	1961 (74.70)	<0.001
Exit to ICU		25 (0.37)	7 (0.17)	18 (0.69)	0.001
Year of Surgery					0.001
	2017	1081 (16.05)	695 (16.90)	386 (14.70)	
	2018	1043 (15.48)	602 (14.64)	441 (16.80)	
	2019	1017 (15.10)	578 (14.06)	439 (16.72)	
	2020	1158 (17.19)	734 (17.85)	424 (16.15)	
	2021	1215 (18.03)	755 (18.36)	460 (17.52)	
	2022	1223 (18.15)	748 (18.19)	475 (18.10)	
Surgery					<0.001
	1st	4833 (71.74)	2736 (66.54)	2097 (79.89)	
	2nd	1498 (22.24)	1176 (28.60)	322 (12.27)	
	Revision	406 (6.03)	200 (4.86)	206 (7.85)	

TKA, total knee arthroplasty; THA, total hip arthroplasty; SD, standard deviation; RBC, red blood cell; FFP, fresh frozen plasma; PLT, platelet; MgSO_4_, magnesium sulfate; DM, diabetes mellitus; HTN, hypertension; CAD, coronary artery disease; CVD, cerebrovascular disease; BMI, body mass index; ASA, American Society of Anesthesiologists; R/A, regional anesthesia; ICU, intensive care unit.

**Table 2:** Comparison of characteristics between two groups: Blood transfusion (BT) vs. Non-BT Group.

Variable		BT Group N = 868	Non-BT Group N = 5869	*p* Value
Age, Year		63.59 (14.68)	66.52 (12.16)	<0.001
Sex, Female		641 (73.85)	4281 (72.94)	0.575
Delirium		29 (3.34)	138 (2.35)	0.080
Estimated Blood Loss (mL/100)		9.38 (8.81)	2.96 (4.21)	<0.001
Anemia		432 (49.77)	1664 (28.35)	<0.001
MgSO_4_		241 (27.76)	1445 (24.62)	0.046
Medical History				
	DM	136 (15.67)	1379 (23.50)	<0.001
	HTN	450 (51.84)	3483 (59.35)	<0.001
	CAD	93 (10.71)	520 (8.86)	0.076
	CVD	95 (10.94)	594 (10.12)	0.455
	Liver Disease	11 (1.27)	23 (0.39)	0.001
	Dementia	6 (0.69)	15 (0.26)	0.032
	Chronic Kidney Disease	37 (4.26)	96 (1.64)	<0.001
	Mental Illness	14 (1.61)	111 (1.89)	0.571
BMI				<0.001
	Underweight	37 (4.26)	51 (0.87)	
	Healthy weight	460 (53.00)	1951 (33.24)	
	Overweight	287 (33.06)	2887 (49.19)	
	Obese	84 (9.68)	980 (16.70)	
ASA Physical Status Classification				<0.001
	1	168 (19.35)	909 (15.49)	
	2	512 (58.99)	4122 (70.23)	
	≥3	188 (21.66)	838 (14.28)	
Anesthesia Time (min/30)		5.66 (1.90)	4.57 (1.19)	<0.001
Type of Anesthesia				<0.001
	Regional Anesthesia	763 (87.90)	5548 (94.53)	
	General Anesthesia	105 (12.10)	321 (5.47)	
Use of Sedatives				
	Dexmedetomidine	189 (21.77)	1060 (18.06)	0.009
	Propofol (R/A only)	308 (35.48)	2608 (44.44)	<0.001
	Midazolam	597 (68.78)	3638 (61.99)	<0.001
Exit to ICU		14 (1.61)	11 (0.19)	<0.001
Year of Surgery				<0.001
	2017	215 (24.77)	866 (14.76)	
	2018	263 (30.30)	780 (13.29)	
	2019	146 (16.82)	871 (14.84)	
	2020	80 (9.22)	1078 (18.37)	
	2021	97 (11.18)	1118 (19.05)	
	2022	67 (7.72)	1156 (19.70)	
Surgery				<0.001
	1st	510 (58.76)	4323 (73.66)	
	2nd	221 (25.46)	1277 (21.76)	
	Revision	137 (15.78)	269 (4.58)	
Type of Surgery				<0.001
	TKA	238 (27.42)	3874 (66.01)	
	THA	630 (72.58)	1995 (33.99)	

MgSO_4_, magnesium sulfate; DM, diabetes mellitus; HTN, hypertension; CAD, coronary artery disease; CVD, cerebrovascular disease; BMI, body mass index; ASA, American Society of Anesthesiologists; R/A, regional anesthesia; ICU, intensive care unit; TKA, total knee arthroplasty; THA, total hip arthroplasty.

**Table 3:** Multivariable logistic regression analysis for delirium after TKA and THA.

Variable		aOR (95% CI)	*p*-Value
Blood Transfusion Group (vs. Non-BT Group; Model 1)		1.03 (0.62, 1.71)	0.917
Blood Transfusion in Detail (Model 2)			
	RBC (vs. No RBC Transfusion Group)	0.88 (0.50, 1.55)	0.651
	FFP (vs. No FFP Transfusion Group)	5.96 (2.72, 13.04)	<0.001
	PLT (vs. No PLT Transfusion Group)	0.00 (0.00)	0.998
	CPP (vs. No CPP Transfusion Group)	0.00 (0.00)	0.999
Estimated Blood Loss		1.01 (0.98, 1.04)	0.423
Anemia		1.60 (1.08, 2.38)	0.019
Medical History			
	CAD	1.60 (0.98, 2.61)	0.058
	CVD	1.38 (0.87, 2.19)	0.167
	Dementia	20.22 (7.53, 54.31)	<0.001
	Chronic Kidney Disease	1.15 (0.49, 2.72)	0.745
	Mental Illness	13.20 (8.12, 21.45)	<0.001
BMI			0.104
	Underweight	2.03 (0.74, 5.56)	0.170
	Healthy weight	1	
	Overweight	0.72 (0.50, 1.03)	0.070
	Obese	0.86 (0.53, 1.38)	0.528
ASA Physical Status Classification			0.169
	1	1	
	2	1.58 (0.87, 2.88)	0.134
	≥3	1.97 (0.97, 4.00)	0.059
Type of Anesthesia			
	Regional Anesthesia	1	
	General Anesthesia	0.62 (0.07, 5.31)	0.661
Use of Sedatives			
	Propofol (R/A only)	0.85 (0.59, 1.21)	0.362
	Midazolam	0.74 (0.52, 1.05)	0.094
Exit to ICU (vs. General ward)		1.57 (0.28, 8.88)	0.612
Year of Surgery			0.314
	2017	1	
	2018	1.66 (0.90, 3.03)	0.102
	2019	0.92 (0.46, 1.84)	0.818
	2020	1.50 (0.81, 2.81)	0.201
	2021	1.38 (0.73, 2.63)	0.323
	2022	1.58 (0.84, 2.97)	0.158
Surgery			0.573
	1st	1	
	2nd	0.83 (0.53, 1.29)	0.400
	revision	1.16 (0.63, 2.14)	0.645

TKA, total knee replacement arthroplasty; THA, total hip replacement arthroplasty; aOR, adjusted odds ratio; CI, confidence interval; FFP, fresh frozen plasma; PLT, platelet; CPP, Cryoprecipitate; CAD, coronary artery disease; CVD, cerebrovascular disease; BMI, body mass index; ASA, American Society of Anesthesiologists; R/A, regional anesthesia; ICU, intensive care unit.

**Table 4:** Subgroup analyses.

BT Group (vs. Non-BT Group)	aOR (95% CI)	*p*-Value
Age > 60 year old	1.14 (0.64, 2.02)	0.658
Age ≤ 60 year old	0.64 (0.19, 2.18)	0.474
Male	0.95 (0.31, 2.94)	0.928
Female	1.08 (0.60, 1.96)	0.796
TKA	1.32 (0.62, 2.83)	0.472
THA	0.65 (0.32, 1.35)	0.250
Anemia group	1.39 (0.72, 2.67)	0.323
Non-anemia group	0.64 (0.25, 1.65)	0.355

BT, blood transfusion; aOR, adjusted odds ratio; CI, confidence interval; TKA, total knee replacement arthroplasty; THA, total hip replacement arthroplasty.

## Data Availability

Data will be available upon reasonable request from the corresponding author.

## Supplementary Materials

The following supporting information can be downloaded at: https://www.mdpi.com/article/10.3390/jpm14060576/s1, Table S1. Univariable Logistic Regression Analyses for Delirium after TKA and THA.

## References

[1] Madry H. (2022). Surgical therapy in osteoarthritis. Osteoarthr. Cartil..

[2] Choi H.J., Yoon H.K., Oh H.C., Yoo J.H., Choi C.H., Lee J.H., Park S.H. (2021). Incidence and risk factors analysis for mortality after total knee arthroplasty based on a large national database in Korea. Sci. Rep..

[3] Wood A.M., Brock T.M., Heil K., Holmes R., Weusten A. (2013). A Review on the Management of Hip and Knee Osteoarthritis. Int. J. Chronic. Dis..

[4] Singh J.A., Yu S., Chen L., Cleveland J.D. (2019). Rates of Total Joint Replacement in the United States: Future Projections to 2020-2040 Using the National Inpatient Sample. J. Rheumatol..

[5] Daugberg L., Jakobsen T., Nielsen P.T., Rasmussen M., El-Galaly A. (2021). A projection of primary knee replacement in Denmark from 2020 to 2050. Acta Orthop..

[6] Frisch N.B., Wessell N.M., Charters M.A., Yu S., Jeffries J.J., Silverton C.D. (2014). Predictors and Complications of Blood Transfusion in Total Hip and Knee Arthroplasty. J. Arthroplast..

[7] Isbister J.P., Shander A., Spahn D.R., Erhard J., Farmer S.L., Hofmann A. (2011). Adverse blood transfusion outcomes: Establishing causation. Transfus. Med. Rev..

[8] Whitlock E.L., Behrends M. (2015). Blood Transfusion and Postoperative Delirium. Curr. Anesthesiol. Rep..

[9] OuYang C.-L., Hao X.-Y., Yu Y., Lou J.-S., Cao J.-B., Yu Y.-Q., Mi W.-D. (2023). Intraoperative allogeneic transfusion is associated with postoperative delirium in older patients after total knee and hip arthroplasty. Front. Surg..

[10] Weinstein S.M., Poultsides L., Baaklini L.R., Morwald E.E., Cozowicz C., Saleh J.N., Arrington M.B., Poeran J., Zubizarreta N., Memtsoudis S.G. (2018). Postoperative delirium in total knee and hip arthroplasty patients: A study of perioperative modifiable risk factors. Br. J. Anaesth..

[11] Yoo S., Lee K.H., Lee H.J., Ha K., Lim C., Chin H.J., Yun J., Cho E.-Y., Chung E., Baek R.-M. (2012). Seoul National University Bundang Hospital’s Electronic System for Total Care. Healthc. Inform. Res..

[12] WHO (2011). Haemoglobin Concentrations for the Diagnosis of Anemia and Assessment of Severity: VMNIS|Vitamin and Mineral Nutrition Information System. Report of a WHO Scientific Group.

[13] Pi-Sunyer F.X., Becker D.M., Bouchard C., Carleton R.A., Colditz G.A., Dietz W.H., Foreyt J.P., Garrison R.J., Grundy S.M., Hansen B.C. (1998). Executive Summary of the Clinical Guidelines on the Identification, Evaluation, and Treatment of Overweight and Obesity in Adults. J. Am. Diet. Assoc..

[14] Lee S.S., Kim J.H., Lee J.J., Kwon Y.S., Seo E.M. (2023). The Impact of Blood Transfusion in Developing Postoperative Delirium in Patients with Hip Fracture Surgery. J. Clin. Med..

[15] Raphael J., Hensley N.B., Chow J., Parr K.G., McNeil J.S., Porter S.B., Taneja M., Tanaka K., Mazzeffi M. (2021). Red Blood Cell Transfusion and Postoperative Delirium in Hip Fracture Surgery Patients: A Retrospective Observational Cohort Study. Anesth. Res. Pract..

[16] Kwon Y.S., Kim J.H., Lee J.J., Seo E.M. (2022). The Relationship between Perioperative Blood Transfusion and Postoperative Delirium in Patients Undergoing Spinal Fusion Surgery: Clinical Data Warehouse Analysis. Medicina.

[17] Andrási T.B., Talipov I., Dinges G., Arndt C., Rastan A.J. (2022). Risk factors for postoperative delirium after cardiac surgical procedures with cardioplegic arrest. Eur. J. Cardio-Thorac. Surg..

[18] Plaschke K., Fichtenkamm P., Schramm C., Hauth S., Martin E., Verch M., Karck M., Kopitz J. (2010). Early postoperative delirium after open-heart cardiac surgery is associated with decreased bispectral EEG and increased cortisol and interleukin-6. Intensive Care Med..

[19] Cata J.P., Wang H., Gottumukkala V., Reuben J., Sessler D.I. (2013). Inflammatory response, immunosuppression, and cancer recurrence after perioperative blood transfusions. BJA Br. J. Anaesth..

[20] Migirov A., Chahar P., Maheshwari K. (2021). Postoperative delirium and neurocognitive disorders. Curr. Opin. Crit. Care.

[21] Clark I.A., Vissel B. (2015). Amyloid β: One of three danger-associated molecules that are secondary inducers of the proinflammatory cytokines that mediate Alzheimer’s disease. Br. J. Pharmacol..

[22] Simmons J.D., Lee Y.L., Pastukh V.M., Capley G., Muscat C.A., Muscat D.C., Marshall M.L., Brevard S.B., Gillespie M.N. (2017). Potential contribution of mitochondrial DNA damage associated molecular patterns in transfusion products to the development of acute respiratory distress syndrome after multiple transfusions. J. Trauma Acute Care Surg..

[23] Liu Y.-M., Huang H., Gao J., Zhou J., Chu H.-C. (2022). Hemoglobin concentration and post-operative delirium in elderly patients undergoing femoral neck fracture surgery. Front. Med..

[24] Zhou Q., Zhou X., Zhang Y., Hou M., Tian X., Yang H., He F., Chen X., Liu T. (2021). Predictors of postoperative delirium in elderly patients following total hip and knee arthroplasty: A systematic review and meta-analysis. BMC Musculoskelet. Disord..

[25] Bilotta F., Lauretta M., Borozdina A., Mizikov V.M., Rosa G. (2013). Postoperative delirium: Risk factors, diagnosis and perioperative care. Minerva Anestesiol..

[26] Steiner L.A. (2011). Postoperative delirium. Part 1: Pathophysiology and risk factors. Eur. J. Anaesthesiol. EJA.

[27] Robinson T.N., Raeburn C.D., Tran Z.V., Angles E.M., Brenner L.A., Moss M. (2009). Postoperative delirium in the elderly: Risk factors and outcomes. Ann. Surg..

[28] Lee H.B., Mears S.C., Rosenberg P.B., Leoutsakos J.-M.S., Gottschalk A., Sieber F.E. (2011). Predisposing Factors for Postoperative Delirium After Hip Fracture Repair in Individuals with and without Dementia. J. Am. Geriatr. Soc..

[29] Aldecoa C., Bettelli G., Bilotta F., Sanders R.D., Audisio R., Borozdina A., Cherubini A., Jones C., Kehlet H., MacLullich A. (2017). European Society of Anaesthesiology evidence-based and consensus-based guideline on postoperative delirium. Eur. J. Anaesthesiol. EJA.

[30] Kosar C.M., Tabloski P.A., Travison T.G., Jones R.N., Schmitt E.M., Puelle M.R., Inloes J.B., Saczynski J.S., Marcantonio E.R., Meagher D. (2014). Effect of preoperative pain and depressive symptoms on the risk of postoperative delirium: A prospective cohort study. Lancet Psychiatry.

[31] Elsamadicy A.A., Adogwa O., Lydon E., Sergesketter A., Kaakati R., Mehta A.I., Vasquez R.A., Cheng J., Bagley C.A., Karikari I.O. (2017). Depression as an independent predictor of postoperative delirium in spine deformity patients undergoing elective spine surgery. J. Neurosurg. Spine SPI.

