# Supplementary material for: Perioperative Blood Transfusion and Delirium after Total Knee or Hip Arthroplasty: Retrospective Analysis
Source: J Pers Med. 2024 May 28;14(6):576. doi: 10.3390/jpm14060576 (PMC11204434; doi:10.3390/jpm14060576)
Supplement: Supplementary file 1 [file jpm-14-00576-s001.zip › jpm-3013037-supplementary.pdf]

Table S1. Univariable Logistic Regression Analyses for Delirium after TKA and THA

Variable	OR (95% CI)	*P*-value
Age, Year	1.01 (1.00, 1.02)	0.108
Female Sex (vs. Male sex)	1.18 (0.82, 1.68)	0.379
Transfusion		
Any	1.44 (0.96, 2.16)	0.082
RBC	1.33 (0.83, 2.13)	0.243
FFP	4.86 (2.62, 9.02)	<0.001
PLT	0.00 (0.00,)	0.998
Cryoprecipitate	0.00 (0.00,)	1.000
Estimated Blood Loss	1.01 (0.98, 1.03)	0.648
Anemia	1.75 (1.28, 2.38)	<0.001
MgSO4	0.98 (0.68, 1.39)	0.886
Medical History		
DM	1.02 (0.70, 1.47)	0.933
HTN	1.25 (0.91, 1.71)	0.177
CAD	2.15 (1.43, 3.24)	<0.001
CVD	2.49 (1.71, 3.63)	<0.001
Liver Disease	2.48 (0.59, 10.42)	0.216
Dementia	25.38 (10.37, 62.09)	<0.001
Chronic Kidney Disease	2.24 (1.03, 4.87)	0.042
Mental Illness	12.74 (8.06, 20.13)	<0.001
BMI		0.093
Underweight	2.08 (0.82, 5.28)	0.125
Healthy weight	1	
Overweight	0.74 (0.53, 1.05)	0.088
Obese	0.90 (0.57, 1.41)	0.638

ASA Physical Status Classification		<0.001
1	1	
2	2.03 (1.14, 3.61)	0.017
≥3	3.48 (1.86, 6.53)	<0.001
Anesthesia Time	0.96 (0.85, 1.08)	0.500
Type of Surgery		
TKA	1	
THA	1.00 (0.73, 1.37)	0.991
Type of Anesthesia		
Regional Anesthesia	1	
General Anesthesia	2.19 (1.37, 3.50)	0.001
Use of Sedatives		
Dexmedetomidine	0.81 (0.53, 1.23)	0.318
Propofol (R/A only)	0.72 (0.51, 1.01)	0.054
Midazolam	0.59 (0.43, 0.80)	0.001
Exit to ICU (vs. General Ward)	3.45 (0.81, 14.76)	0.095
Year of Surgery		0.128
2017	1	
2018	1.87 (1.04, 3.35)	0.036
2019	1.06 (0.55, 2.06)	0.853
2020	1.62 (0.90, 2.92)	0.105
2021	1.50 (0.83, 2.70)	0.182
2022	1.89 (1.07, 3.34)	0.027
Surgery		0.173
1st	1	
2nd	1.20 (0.83, 1.72)	0.331
Revision	1.63 (0.94, 2.83)	0.080

TKA total knee replacement arthroplasty; *THA* total hip replacement arthroplasty; *OR* odds ratio; *CI* confidence interval; *RBC* red blood cell; *FFP* fresh frozen plasma; *PLT* platelet; *MgSO4* magnesium sulfate; *DM* diabetes mellitus; *HTN* hypertension; *CAD* coronary artery disease; *CVD* cevebrovascular disease; *BMI* body mass index; *ASA* American Society of Anesthesiologists; *R/A* regional anesthesia; *ICU* intensive care unit
